# Botanical Description of British Plants

**Published:** 1807-11

**Authors:** 


( To be continued. )

---

### *Botanical Description of British Plants.*

[ Continued from Vol. xviii. page 363—371. ]

Monadelphia. Polyandria.

2. Althæa. *A. officinalis.—A. Ibiscus. A. sive Bismalva.*

*Ang.* Marsh mallow. Wymote.

*Gen. Desc.* Cal. double; outer nine-cleft. Capsules many, one seed in each.

*Spec. Desc.* *Leaves* undivided, angular, cottony, obscurely lobed, doubly serrated, *lower* egg-shaped, *upper* egg-spear-shaped; very soft like velvet, on leaf stalks. *Stem* upright, a yard high, cylind. velvety, branched. *Flowers* in panicles, axillary, on fruit-st.; white, or pale flesh colour. *Pet.* fringed at base, nicked. Cal. sometimes eleven or twelve cleft.—*Salt marshes and banks of rivers.* Bl. Aug.

---

*Use.* The dry roots, boiled in water, give out half their weight of a gummy matter, (nearly allied to gum arabic, tragacanth, starch, &c. &c. dissolving myrrh and other resinous substances, more readily than the first.—*Buckholz)* which, on evaporating the aqueous fluid, forms a flavorless yellow mucilage. This mucilaginous matter is the medicinal part of the plant, and is commonly employed for its emollient and demulcent qualities: its use is recommended where the natural mucus of membranes becomes acrid or abraded; for obtunding and incrassating acrimonious thin fluids, in tickling coughs from the defluxions of the fauces and lungs, in hoarseness, erosions of the stomach and intestines, strangury, and for lubricating and relaxing the passages in nephritic and calculous complaints.—*Lewis.* It is directed only in the form of a syrup: the root was formerly used in substance.—*Woodville.* The root and leaves have a mucilaginous quality, and are often used in a syrup or decoction as a balsamic pectoral for coughs and hoarsenesses. It is also found useful in nephritic complaints and the strangury: and is employed in cataplasms and fomentations against swellings.—The root will turn water to a jelly.—*Lightfoot.* The root boiled is much used as an emollient cataplasm; and an infusion of it is very generally prescribed in all cases where mild mucilaginous substances are useful.—*Withering.*

3. MALVA. *M. sylvestris.*—*M. vulgaris.*

*Ang.* Common mallow. Mauls.

*Gen. Desc.* Cal. double; outer mostly three leaved. Caps. eight or more in a whirl; one, rarely two-celled; one seed in each.

*Spec. Desc.* *Stem* rough, hairy, rather woody. *Leaves* five or seven lobed, toothed, hairy, a dark purple stain near the insertion of the leaf stalk. *Outer cal.* leafits partly united at the base: *inner* and *outer* hairy without, smooth within, toothed at the edges. *Fruit-st.* cylind. axillary. *Petals* nicked, purple or bluish, with three or four darker streakes. *Hedges, footpaths, rubbish.* BL. June, Aug.

*Use.* This plant has a strong affinity to althæa both in a botanical and medicinal respect: but the leaves and flowers only are directed for use; the leaves afford a similar glutinous juice, fitted to answer the same purposes; and are therefore principally used in fomentations, cataplasms, and emollient enemas, but their internal use is superseded by the root of the althœa. Formerly, in the infancy of gardening, gardening, when the choice of esculent plants was very limited, this was a common article of diet: and the Chinese still eat the leaves, either raw as sallad, or boiled like spinage.—*Woodville.* A decoction of the whole plant, which is mucilaginous and emollient, or an infusion of the flowers, is recommended as a pectoral, and good for the stone and gravel, and other complaints in the urinary passages. It is likewise given in clysters in the dysentery, tenesmus, and gonorrhœa; and is used by way of cataplasm in inflammations.—*Lightfoot.*

4. Taxus. *T. baccata.*

*Ang.* Yew tree.

*Gen. Desç.* Diæcious. Bloss. 0. Cal. a four or a seven-leaved bud. M. anthers target-shaped, eight cleft. F. style 0. Seed one, surrounded at the base by a pulpy receptacle; the upper half naked.

*Spec. Desc. Leaves* solitary, strap-shaped, prickle-pointed, near together. *Recept.* of M. fl. somewhat globular. *Berries* proceeding from a recept. which half covers and protects the seed, red when ripe *Seeds* with two seed-lobes.—*Mountains, woods.* Bl. March, April.

*Use.* The berries are sweet and viscid: children often eat them in large quantities without any inconvenience: but the fresh leaves are fatal to the human species; three children were killed by a spoonful of the green leaves; they died without agony or any of the usual symptoms of vegetable poisons; the same quantity of the dried leaves had been given the day before without any effect. *Percival's Ess. iii.* And though it is eaten by sheep and goats, there are instances of both being killed by it, branches having been found in their stomachs. *Gent. Mag.* lvi. 941. Sheep are said to have been killed by browsing on the bark: the loppings, in a half dried state, Dr. Withering thinks the most detrimental to cattle. The wood is hard, smooth, and beautifully veined with red: it is converted into bows, axles, spoons, cogs for mill wheels, and flood gates for fish ponds, which hardly ever decay. It grows best in a moist loamy soil: on bogs or dry mountains it languishes. It bears transplanting even when old; and as it bears clipping, it is often used for hedges, which form excellent skreens to keep off the cold winds from tender plants. The berries are eaten by swine and field fares; the leaves by sheep and goats; horses and cows refuse them.—*Withering.*

5. Pinus. *P. sylvestris.—Pix liquida.*
*Ang.* Scotch fir.
*Gen. Desc.* Monæcious. Bloss. 0. M. calyx scales forming a bud standing open. Anthers naked. F. cal. scales forming a cone, two flowers in each scale. Pistil one. Nut of one cell, without valves, bordered with a membrane.
*Spec. Desc.* *Leaves* in pairs, rigid. *Cones* egg-shaped, mostly in pairs, as long as the leaves, whitish, pendant. *Scales* oblong, blunt.—*Highland mountains.* Bl. May.
*Use.* This tree furnishes most abundantly the *Pix liquida*, or tar; and from it also may be obtained the common turpentine, and the white and yellow resins. Tar is procured by cutting the tree into pieces, which are inclosed in a large oven, constructed for the purpose with a channel at the bottom: by the application of sufficient heat, the tar is forced out of the wood, and runs off by the channel at the bottom. Tar, well known for its œconomical uses, is properly an empyreumatic oil of turpentine; and has been much used as a medicine, internally and externally: water impregnated with its more soluble parts, called tar-water, was some time ago a very popular remedy for various disorders both acute and chronic; and, though its efficacy was much exaggerated by Bp. Berkeley and others, Dr. Cullen found it in several cases a valuable medicine; and says, it appeared to strengthen the tone of the stomach, to excite appetite, promote digestion, and cure all symptoms of dyspepsia: at the same time it promotes the excretions, particularly that of urine, so that it is obvious that in many disorders this medicine may be highly useful. *M. M. ii.* 334. The common proportions are 2 ℔ of tar to 1 gallon of water, well stirred together, then suffered to settle for two days and the clear liquor poured off; from a pint to a quart of it, according to circumstances, may be taken in the course of twenty-four hours.—*Woodville.* The inhabitants of the North of Europe use the bark for making bread, which they do in the following manner: they choose a tree whose trunk is even, (for these contain the least resin), and strip off the bark in the spring when it separates most readily: this they first dry gently in the shade; then in a greater heat; then reduce it to powder; with which they mix a small quantity of corn meal, and with water knead it into bread: this they eat, not only in years of scarcity, but at other times, from an apprehension that long disuse might render it disagreeable to them. Their children are very fond of the fresh bark in the spring time, time, either shaved with a knife, or grated with a rasp. The young shoots distilled afford a fragrant essential oil.—The bark will tan leather. This tree furnishes the best red or yellow deal; it is smooth, light, and easily cloven. It flourishes best in a poor sandy soil: on rocks or bogs it seldom attains a large size; near stagnant water it never thrives; in black soil it becomes diseased, and in chalky land it dies; it will not bear the least clipping because none but the terminating buds send forth branches. It suffers by transplanting, because the central root grows perpendicularly downwards, and if this be broken or interrupted in its passage, the stem ceases to shoot upwards, and the tree remains a dwarf; the other roots spread near the surface. *Withering.*

From this tree, by incision, barras, burgundy pitch, and turpentine are acquired and prepared. The resinous roots dry out of the ground, and divided into small splinters are used in many parts of the Highlands of Scotland, to burn instead of candles. An infusion or tea of the buds is highly recommended as an antiscorbutic. From the cones is prepared a diuretic oil; like the oil of turpentine and a resinous extract, which have virtues similar to those of the Balsam of Peru. In Rossshire the inner bark is used by fishermen for making ropes; and of it also, bread is made by the inhabitants of Sweden, Lapland, and Kamschatka: the Swedish boys frequently tear off this inner bark in the spring and eat it with a greedy appetite. It serves also to fatten swine. It is said by Linnæus that this tree lives to the age of 400 years.—*Lightfoot.* Neither horses, sheep, nor goats are fond of it.

( To be continued. )

